# Ginsenoside Rb1 Alleviated High-Fat-Diet-Induced Hepatocytic Apoptosis via Peroxisome Proliferator-Activated Receptor *γ*

**DOI:** 10.1155/2020/2315230

**Published:** 2020-07-13

**Authors:** Bing Song, Yao Sun, Yafen Chu, Jing Wang, Hongwei Zheng, Lili Liu, Wang Cai, Haoqiang Zhang

**Affiliations:** ^1^Department of Endocrinology, First Affiliated Hospital of Jinzhou Medical University, China; ^2^Department of Pharmacy, Taikang Xianlin Drum Tower Hospital, Medical School of Nanjing University, China; ^3^Department of Endocrinology, Ningbo Medical Center Lihuili Hospital, China; ^4^Department of Obstetrics and Gynecology, First Affiliated Hospital of Jinzhou Medical University, China; ^5^Department of Endocrinology, Affiliated Zhongda Hospital of Southeast University, China

## Abstract

**Objective:**

High-fat-diet- (HFD-) induced hepatic cell apoptosis is common in mice with nonalcoholic fatty liver disease (NAFLD). We aim to investigate the effect of Ginsenoside Rb1 (GRb1) on hepatocyte apoptosis.

**Methods:**

C57BL/6J mice with HFD were used to induce a liver-injured model with cell apoptosis. In addition, GRb1 was used to treat HFD-induced apoptosis in a liver with or without inhibitor of peroxisome proliferator-activated receptor *γ* (PPAR-*γ*).

**Results:**

Compared with C57BL/6J mice with common chow, there are downregulated PPAR-*γ* but upregulated cell apoptosis in the liver of mice with HFD. Furthermore, GRb1 elevated the hepatic PPAR-*γ* level and suppressed hepatocytic apoptosis. However, GW9662 abolished the effects of GRb1 on apoptosis in the liver.

**Conclusions:**

GRb1 alleviated HFD-induced apoptosis of hepatocytes of mice via PPAR-*γ*.

## 1. Introduction

With a prevalence of 25–30% in the general population [1] and 3–12% in children [2], NAFLD is one of the most common chronic liver diseases throughout the world [3]. Obesity and other metabolic syndrome features, such as insulin resistance (IR), hyperglycemia, and hyperlipidemia, are well-considered risk factors for NAFLD. In addition, a recent study suggested that NAFLD prevalence is increasing especially in obese children (ranging from 70 to 90%) [4]. Furthermore, NAFLD in obese individuals, with systemic low-grade chronic inflammation status and IR, is a characteristic of cell apoptosis, especially in livers of patients with nonalcoholic steatohepatitis (NASH) [5].

Damage-associated molecular patterns (DAMPs) are endogenous molecules released by stressed cells in response to tissue injury. DAMPs may cause liver necroinflammation and fibrosis in NAFLD [6]. High-mobility group box 1 protein (HMGB1) was initially described as a late mediator of sepsis due to its release from necrosis of cells. Its role in sterile inflammation was subsequently recognized [7]. As a ligand for Toll-like receptors (TLRs), HMGB1 is a kind of DAMPs that serves to activate innate immunity [8] and trigger apoptosis. Additionally, HMGB1 is actively secreted resulting from cell death (including necrosis and severe apoptosis) [9, 10]. To exert these activities, HMGB1 must be transmitted from the nucleus to the outside of cells through the cytoplasm. In our previous study, we determined that apoptosis is irritated resulting from free fatty acid-activated myd88-dependent TLR2 signaling pathway and downstream inflammatory factors [11]. Although limited apoptosis is not the reason for severe inflammation, necrosis and high level of apoptosis may result in low-grade inflammation and the release of HMGB1. Moreover, released HMGB1 may cause more cell apoptosis. In general, there may be a “HMGB1-apoptosis cycle” in patients with NAFLD, especially in NASH.

PPAR-*γ* is a target of insulin sensitivity, involved in obesity, NAFLD, NASH, and type 2 diabetes mellitus (T2DM) [12]. In recent studies, IR is linked to cell apoptosis in the liver of NAFLD [13]. Pioglitazone, a kind of thiazolidinediones, which is a classic ligand for PPAR-*γ*, plays an essential role in the treatment of IR in T2DM. For the costs and side effect, the development of inexpensive and safe drugs is imperative. In our previous work, emodin, a major anthraquinone derivative that is obtained from Rheum palmatum and some other Chinese herbs [14], increased insulin sensitivity [15] which may result from binding to PPAR-*γ* [16–18]. GRb1 isolated from ginseng, another traditional Chinese medicine, has been proven to have therapeutic effects on treating obesity and diabetes [19–21]. Additionally, GRb1 is involved in IR via 11beta-hydroxysteroid dehydrogenase type I in our previous study [22] and NAFLD in other's study [23]. Moreover, GRb1 showed the ability to activate PPAR-*γ* [24] and be involved in the regulation of IR [25].

However, the role of GRb1 in the progress of hepatocytic apoptosis in NAFLD remains unclear. In the present study, we aim to determine the effects of GRb1 on apoptosis in HFD-induced NAFLD and investigate the roles of PPAR-*γ* and HMGB1 in this process.

## 2. Materials and Methods

### 2.1. Animal Housing and Treatment

Healthy male C57BL/J mice (*n* = 32) at 7 weeks of age were purchased from HFK Bioscience Co., Ltd. (Beijing, China). Prior to animal experiments, all mice were housed in specified-pathogen free for 1 week. The animals were randomly divided into two groups with normal diet or HFD (with 60% fat). After 16 weeks of feeding, the mice with HFD group were administrated with GRb1 (10 mg/kg) [22] (cat no. SG8260; Solarbio Science & Technology Co., Ltd., Beijing, China) with or without GW9662 [26] (4 mg/kg) (cat no. HY-16578; MedChemExpress, Monmouth Junction, NJ, USA) every other day for 8 weeks. The mice with normal chow received saline of the same frequency and the same volume. According to the diet and treatment described above, the animals were divided into 4 groups: normal diet group (ND, *n* = 8), HFD group (HFD, *n* = 8), GRb1 treatment group (HFD-GRb1 or GRb1, *n* = 8), and GRb1 combined with GW9662 group (GRb1-GW9662, *n* = 8). All experiments were performed according to the guidance of the Ethics Committee for Experimental Research from the First Affiliated Hospital of Jinzhou Medical University.

### 2.2. Physiological Assessments

Body weights were measured weekly. One week before the end of the experiment, intraperitoneal glucose tolerance tests (IPGTT) were performed. For IPGTT, mice were fasted for 8 hours and injected with glucose (2 g/kg, i.p.). Blood glucose levels were measured at 30, 60, 90, and 120 minutes after injection of glucose. At the end of the experiment, mice were sacrificed by cervical dislocation. Blood samples were collected to measure the levels of triglycerides (TG), total cholesterol (TC), low-density lipoprotein cholesterol (LDL), and high-density lipoprotein cholesterol (HDL) with a special kit (cat no.: GM1114, GM1113, GM1116, and GM1115, Servicebio, Wuhan, China). Adipose tissues around the epididymis, kidney, and pericardium were isolated and weighed. Livers were stored for further research in a -80°C refrigerator or paraformaldehyde (4%).

### 2.3. Oil Red O Stain

After liver tissue storage in paraformaldehyde for 3 days, livers were embedded in OCT and trimmed to 10 *μ*m sections. Oil red O stain was performed according to the protocol described previously [27].

### 2.4. Cell Preparation and Protein Separation Assay

Cells were prepared as the protocol described previously [28]. Fresh liver tissues isolated from mice with or without HFD, GRb1, and GW9662 were digested by collagenase for 30 min at 37°C. Then, cells were filtered and centrifuged. Cells were used for protein extraction from the nucleus and cytoplasm with a kit (cat no. KGP1100; KeyGEN BioTECH Corp., Ltd., Jiangsu, China) according to the manufacturer's protocol.

### 2.5. Caspase 3 Activity Assay

Total proteins from cells were extracted using radioimmunoprecipitation (RIPA) assay buffer (cat no. wla016a; Wanleibo Co., Ltd., Shenyang, China) according to the protocol of the manufacturer. Subsequently, a BCA assay was performed to measure protein concentration in middle extracting solution. And then, the protein concentration was modulated to 5 mg/ml. Caspase 3 activity was measured by a caspase 3 spectrophotometric detection kit (cat no. wla047; Wanleibo Co., Ltd., Shenyang, China).

### 2.6. Western Blotting

Normal protein extraction and measurement administration were performed as previously by RIPA. However, the protein for HMGB1 measurement was isolated by the kit described above with the kit to get the protein from the nucleus and cytoplasm, respectively. Proteins were separated by SDS-PAGE on 10% gels, transferred to polyvinylidene fluoride membranes, and blocked in milk for 2 h at room temperature. Rabbit anti-mouse primary antibodies to PPAR-*γ* (1 : 800; cat no. wl0269; Wanleibo Co., Ltd., Shenyang, China), HMGB1 (1 : 500; cat no. wl03023; Wanleibo Co., Ltd., Shenyang, China), BAX (1 : 1000; cat no. wl03315; Wanleibo Co., Ltd., Shenyang, China), Bcl2 (1 : 500; cat no. wl01556; Wanleibo Co., Ltd., Shenyang, China), and *β*-actin (1 : 1000; cat no. wl01845; Wanleibo Co., Ltd., Shenyang, China) were used to bind target proteins at 4°C overnight. Following incubation with goat anti-rabbit secondary antibody (1 : 5000; cat no. wla023; Wanleibo Co., Ltd., Shenyang, China) for 2 h at 20-25°C, an enhanced chemiluminescence kit (cat no. wla006a; Wanleibo Co., Ltd., Shenyang, China) was used to detect protein expression. All protein measurements were repeated at least 3 times.

### 2.7. Statistics

All values were described as the mean ± standard deviation. All data were analyzed by one-way ANOVA, for the variance between multiple groups, followed by LSD to compare 2 groups. It was considered as significant if *P* < 0.05.

## 3. Results

### 3.1. Effects of GRb1 on Weight of Body and Adipose Tissue

**Figure 1:**
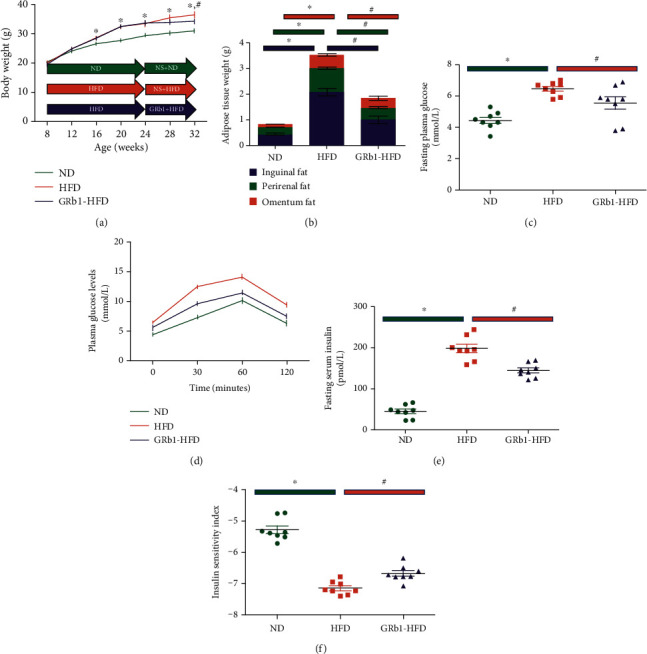
Effects of GRb1 on HFD-induced obesity and insulin resistance. ND: normal diet; HFD: high-fat diet; GRb1-HFD: high-fat-diet mice with GRb1 treatment. ^∗^*P* < 0.05, HFD vs. ND; ^#^*P* < 0.05, GRb1-HFD vs. HFD.

To investigate the effects of GRb1 on obese mice with NAFLD, C57BL/6J mice were fed with HFD. Compared to mice with normal diet, body weight of mice increased sharply after 4 weeks (aged 12 weeks) of HFD feeding. In addition, HFD elevated body weight significantly from 8 weeks (aged 16 weeks) of feeding. However, at the time of 16 weeks (aged 24 weeks), there is no such acute body weight increase. At that time, GRb1 were used to treat HFD-induced mice with NAFLD. Interestingly, compared to mice without GRb1, GRb1 prevented body increase from HFD after 8 weeks of GRb1 treatment (Figure 1(a)). To further explore the effects of GRb1 on lipid metabolism of HFD-induced mice with obesity, adipose tissues (inguinal fat, perirenal fat, and omental fat) were isolated and weighed. Compared with mice with normal diet, HFD increased inguinal, perirenal, and omental adipose tissue weights. However, GRb1 stopped all these three parts of adipose tissue from expanding (Figure 1(b)).

### 3.2. GRb1 Improved the Glucose Metabolism

IR plays an essential role in impaired glucose metabolism and obesity-associated NAFLD. To further investigate the effect of GRb1 on glucose metabolism, fasting plasma glucose and fasting serum insulin were detected; the insulin sensitivity index was calculated prior to the administration of IPGTT. Plasma glucose levels were observed after 30, 60, and 120 minutes of IPGTT. HFD increased fasting plasma glucose (Figure 1(c)), fasting serum insulin (Figure 1(e)), and area under the curve (Figure 1(d)), while decreasing the insulin sensitivity index (Figure 1(f)). Interestingly, GRb1 downregulated the levels of fasting plasma glucose and fasting serum insulin, decreased the area under the curve, and improved insulin resistance.

### 3.3. GRb1 Alleviated Systemic Lipid Metabolism and the Deposition of Lipid in Liver

**Figure 2:**
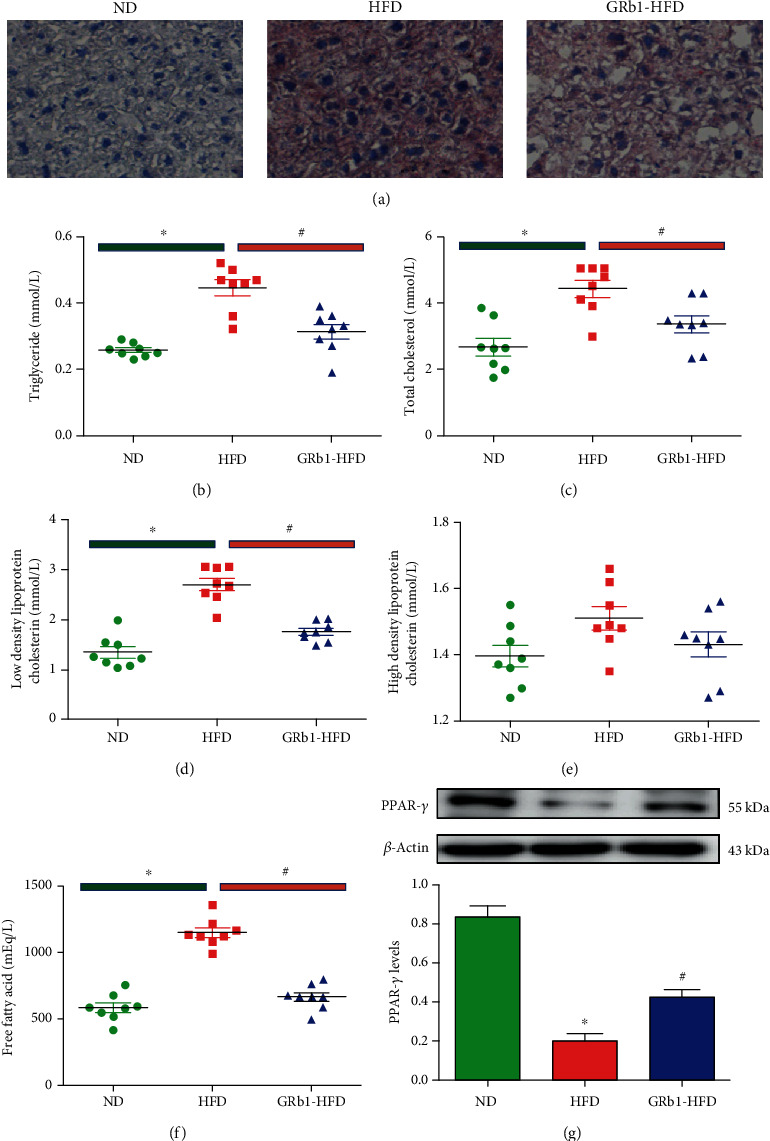
Effects of GRb1 on HFD-induced obesity and insulin resistance. ND: normal diet; HFD: high-fat diet; GRb1-HFD: high-fat-diet mice with GRb1 treatment. ^∗^*P* < 0.05, HFD vs. ND; ^#^*P* < 0.05, GRb1-HFD vs. HFD.

Lipid metabolism is impaired in animal models and humans with obesity and NAFLD. Oil red O staining was performed. We detected increased lipid deposition in mice with HFD (Figure 2(a)). In addition, we demonstrated upregulation of TG (Figure 2(b)), TC (Figure 2(c)), LDL (Figure 2(d)), and free fatty acid (Figure 2(f)) in obese mice with NAFLD. Furthermore, GRb1 treatment administration was performed. Interestingly, GRb1 not only decreased local lipid deposition in the liver but also downregulated global levels of TG, TC, LDL, and free fatty acid. Although no significance was showed on the levels of HDL in statistics, there remains an upregulated HDL in mice with HFD (Figure 2(e)).

### 3.4. GRb1 Increased PPAR-*γ* Levels in Liver

PPAR-*γ* involved in the pathogenesis of NAFLD is an important therapeutic target of IR. To explore the role of PPAR-*γ* in the process of GRb1 alleviating lipid metabolism in the liver of mice with HFD-induced NAFLD, PPAR-*γ* levels were measured. Compared with mice with common diet, we detected decreased PPAR-*γ* in the liver of mice with HFD. However, we observed about 2-fold PPAR-*γ* increase in HFD-feeding mice with GRb1 treatment (Figure 2(g)).

### 3.5. GRb1 Protected Hepatocytes from Apoptosis

**Figure 3:**
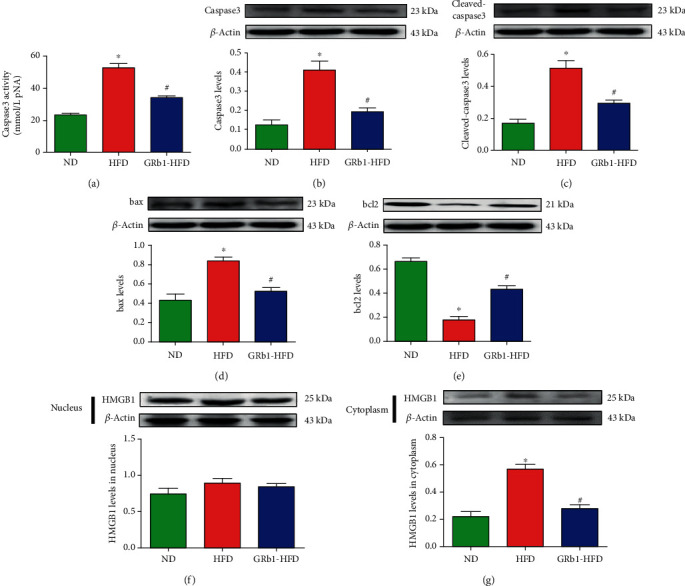
Effects of GRb1 on HFD-induced apoptosis and HMGB1 expression. ND: normal diet; HFD: high-fat diet; GRb1-HFD: high-fat-diet mice with GRb1 treatment. ^∗^*P* < 0.05, HFD vs. ND; ^#^*P* < 0.05, GRb1-HFD vs. HFD.

Free fatty acid-stimulated low-grade inflammation is associated with cell apoptosis and necrosis. To show the effects of GRb1 on hepatocytic apoptosis, livers of normal chow mice and HFD mice with or without GRb1 were isolated to measure the caspase 3 activity and levels of caspase 3, cleaved-caspase 3, bax, and bcl2. To further research the effects of GRb1 on cell apoptosis, caspase 3 activity as well as levels of caspase 3, cleaved-caspase 3, bax, and bcl2 was analyzed. Indeed, we measured upregulated caspase 3 activity and caspase 3 and bax levels and downregulated bcl2 in the mouse liver with HFD. However, there were suppressed caspase 3 activity and caspase 3, cleaved-caspase 3, and bax levels monitored while elevating the bcl2 level in mice with GRb1 (Figures 3(a)–3(e)).

### 3.6. GRb1 Decreased HMGB1 in Cytoplasm of Hepatocytes

HMGB1, in the nucleus, is essential for the growth of cells, while releases to extracellular space through cytoplasm result from the necrosis of cells. It also results in cellular apoptosis and necrosis in turn. To investigate the role of HMGB1 in the protective effects of GRb1 on NAFLD, HMGB1 levels were observed in cytoplasm and nucleus. We observed an upregulated HMGB1 in liver cytoplasm of HFD mice and downregulated HMGB1 in liver cytoplasm of mice with HFD and GRb1. However, there was no significant difference of HMGB1 levels in the liver nucleus of mice with or without HFD and GRb1 (Figures 3(f) and 3(g)).

### 3.7. PPAR-*γ* Inhibitor Abolished the Protective Effects of GRb1 from Lipid Deposition in the Liver as well as Hepatocytic Apoptosis

**Figure 4:**
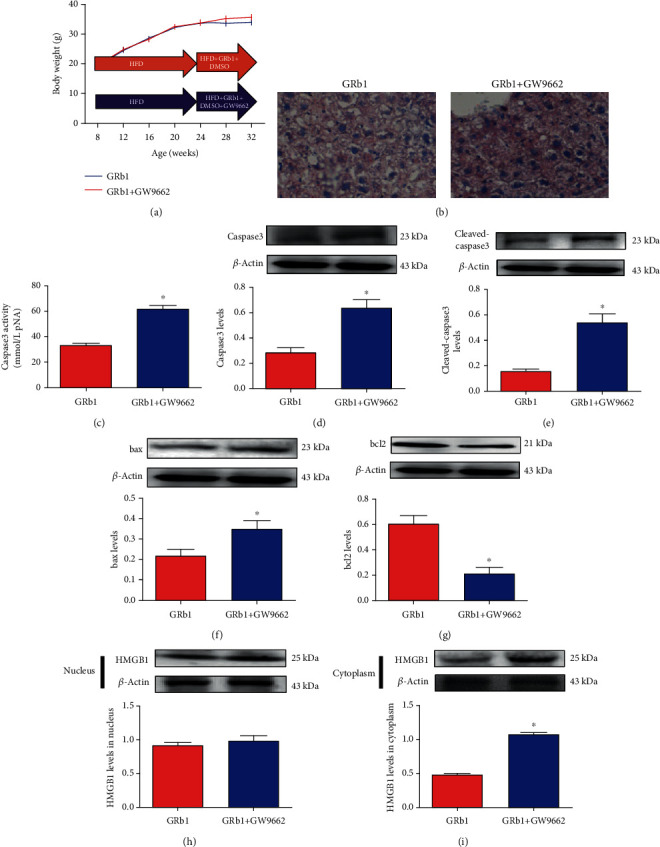
Effects of GRb1 on HFD-induced obesity and insulin resistance. ND: normal diet; HFD: high-fat diet; GRb1-HFD: high-fat-diet mice with GRb1 treatment. ^∗^*P* < 0.05, HFD vs. ND; ^#^*P* < 0.05, GRb1-HFD vs. HFD.

As increased PPAR-*γ* levels decreased by GRb1 were detected in the liver of HFD mice, we guess that PPAR-*γ* may play an important role in the progress of NAFLD. To investigate the essential role of PPAR-*γ* in hepatocyte apoptosis, GW9662 was administrated. GW9662 elevated lipid deposition in the liver of HFD mice with GRb1 (Figure 4(b)). In addition, GW9662 upregulated caspase 3 activity and caspase 3, cleaved-caspase 3, and bax levels but downregulated bcl2 levels (Figures 4(c)–4(g)).

### 3.8. PPAR-*γ* Inhibitor Elevated HMGB1 in Hepatocytic Cytoplasm of Mice with HFD and GRb1

To further explore the role of HMGB1 in the progress of apoptosis, HMGB1 levels in the nucleus and cytoplasm were monitored after the GW9662 administration assay. Though no significance was detected in the hepatocytic nucleus in HFD mice with or without GW9662 (Figure 4(h)), we found increased HMGB1 in cytoplasm in mice with GW9662 (Figure 4(i)).

## 4. Discussion

GRb1, with therapeutic effects on treating obesity and diabetes, is isolated from ginseng, a kind of herb of Chinese traditional medicine [29, 30]. In addition, it is reported to increase insulin sensitivity in HFD mice with NAFLD in our previous study [22]. To explore the effects of GRb1 on NAFLD, HFD mice were administrated with GRb1. Interestingly, GRb1 decreased body and adipose tissue weight of mice with HFD, as well as local lipid metabolism and systemic insulin sensitivity, which is supported by our and other's previous studies [22, 29, 30].

Hepatocytic apoptosis is triggered by increased local and global chronic inflammation, which is a well-recognized characteristic of NAFLD [31]. However, the effect of GRb1 on hepatocytic apoptosis in NAFLD remains unknown. To probe its protective effect, apoptosis was measured by a caspase 3 activity kit and western blotting. It is suggested that apoptosis triggered by HFD was alleviated by GRb1.

HMGB1 is one of the essential components of the nucleus. But it can cause stress when released outside the cell. In this process, it needs to go through the cytoplasm [32]. Although the effects of apoptosis on HMGB1 release are still unclear in NAFLD, extracellular HMGB1 from the nucleus via cytoplasm could lead to chronic inflammation resulting from DAMPs, like free fatty acid. Additionally, exacerbated inflammation is associated with cell apoptosis [31]. To uncover the role of HMGB1 involved in the progress of apoptosis of NAFLD, HMGB1 were measured in the cytoplasm and nucleus of the liver. Interestingly, increased HMGB1 was detected in cytoplasm and not in the nucleus. It suggests that HMGB1 in cytoplasm may play an important role in NAFLD.

PPAR-*γ*, which is involved in insulin sensitivity of NAFLD [12], and apoptosis in other models [33, 34], could be upregulated and activated by GRb1 [24, 35]. In our previous study, we found that GRb1 alleviated insulin resistance in HFD-induced obese mice [22]. To investigate the role of PPAR-*γ* which is involved in hepatocytic apoptosis and necrosis in NAFLD, PPAR-*γ* levels were measured in the liver of mice. Increased PPAR-*γ* levels were observed in mice with HFD but decreased in mice with HFD and GRb1.

To further research the potential role of PPAR-*γ*, GW9662, a classic PPAR-*γ* inhibitor, was administrated to mice with HFD and GRb1. We monitored increased apoptosis and necrosis levels in the liver of mice with GRb1 and GW9662 compared with mice with GRb1. Furthermore, GW9662 upregulated the levels of HMGB1 in cytoplasm, while having no effect on its levels in the nucleus in the liver. These results claim that GRb1 could alleviate HMGB1-induced hepatocytic apoptosis in NAFLD. In addition, PPAR-*γ* plays a very important role in this progress.

## 5. Conclusion

Ginsenoside Rb1 alleviated the HFD-induced cycle of “HMGB1-apoptosis” in the liver of mice. Additionally, this may be partly dependent on PPAR-*γ*. The activation of PPAR-*γ* may be a novel method for the treatment of NAFLD.

## Data Availability

The data used to support the findings of this study are available from the corresponding authors upon request.
